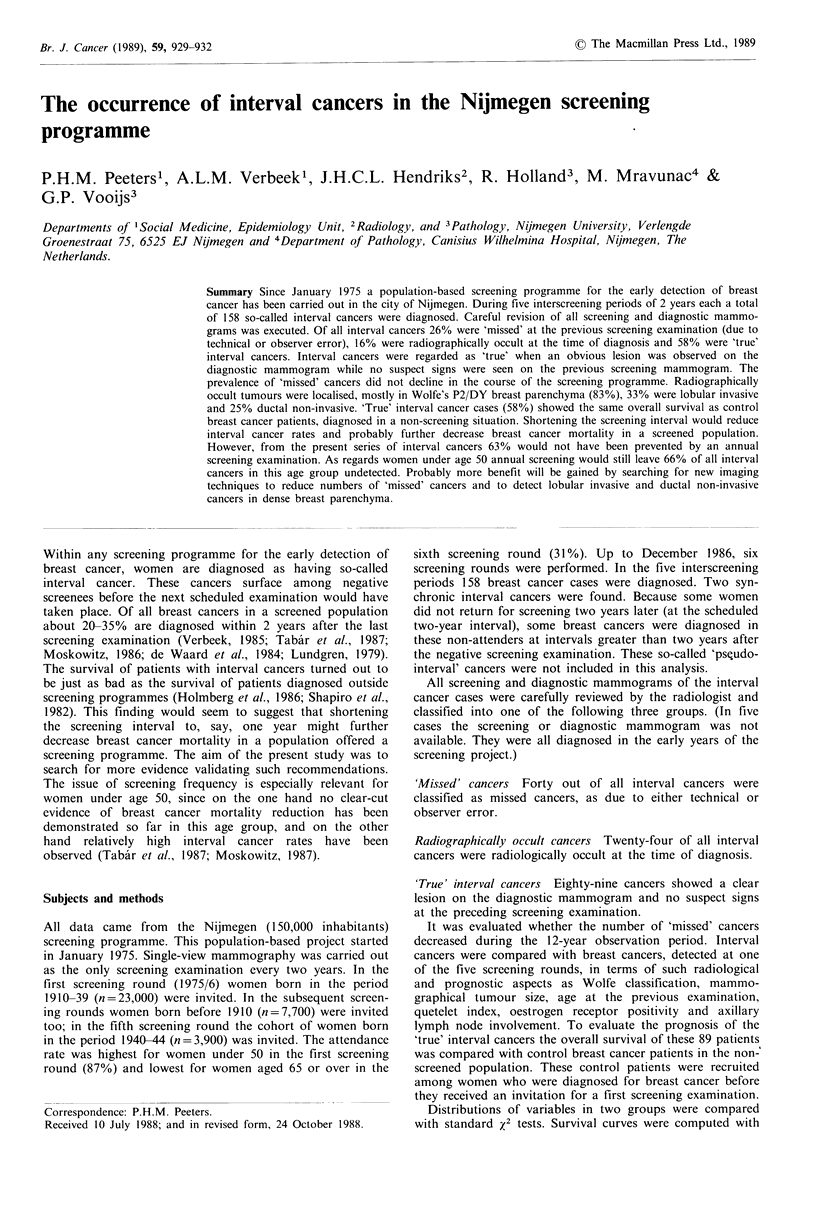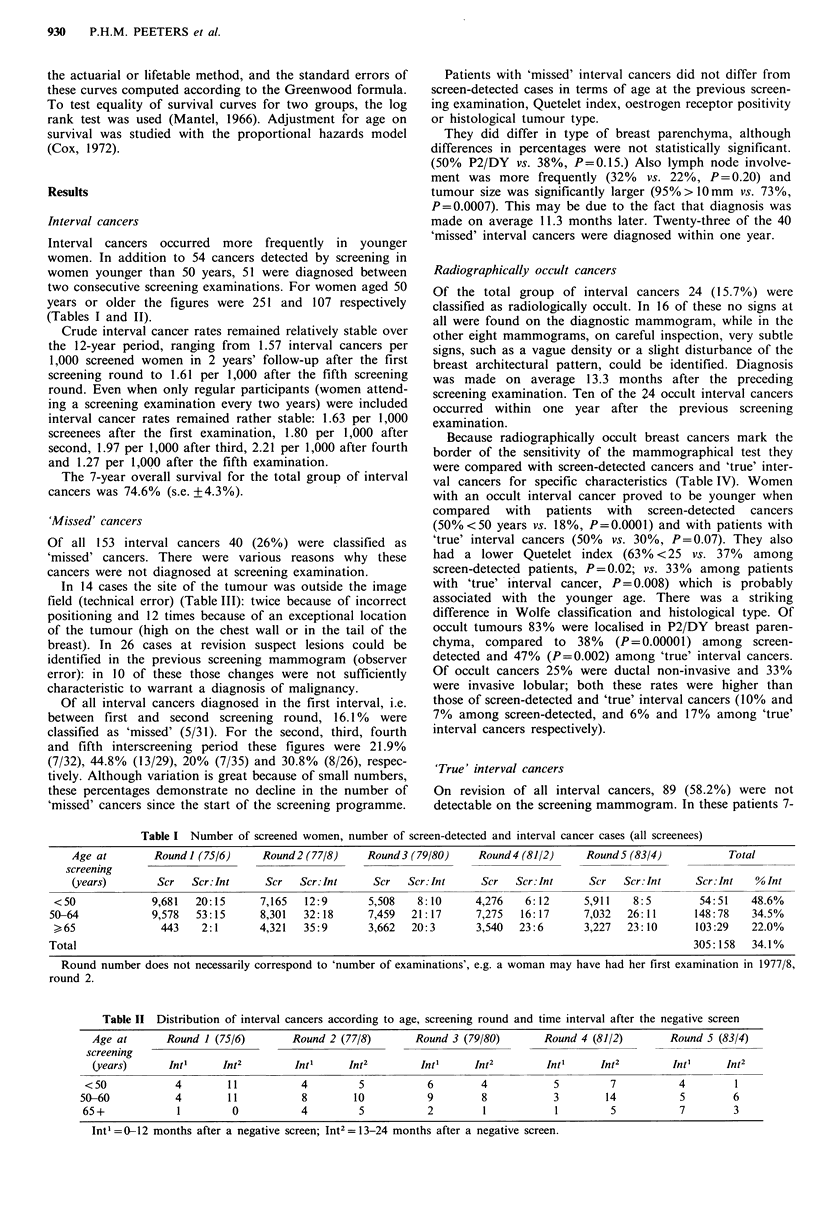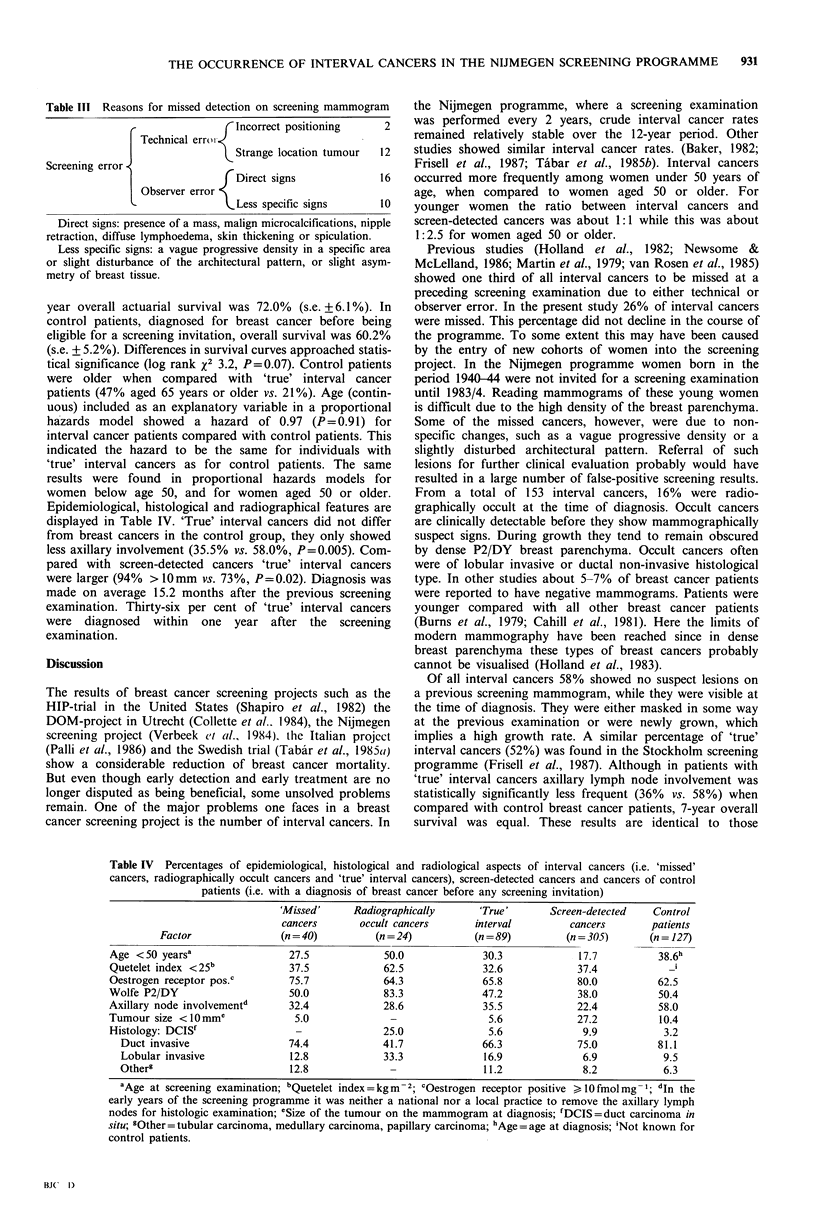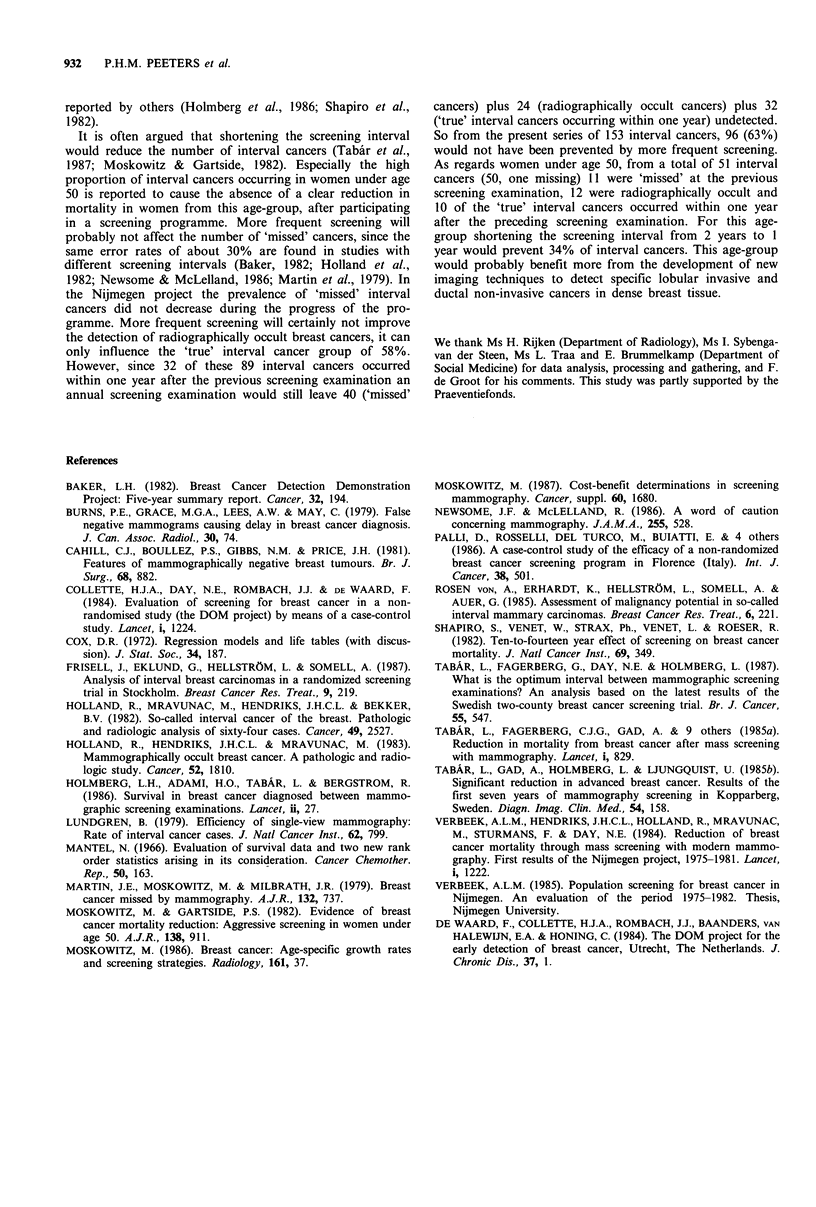# The occurrence of interval cancers in the Nijmegen screening programme.

**DOI:** 10.1038/bjc.1989.196

**Published:** 1989-06

**Authors:** P. H. Peeters, A. L. Verbeek, J. H. Hendriks, R. Holland, M. Mravunac, G. P. Vooijs

**Affiliations:** Department of Social Medicine, Nijmegen University, The Netherlands.

## Abstract

Since January 1975 a population-based screening programme for the early detection of breast cancer has been carried out in the city of Nijmegen. During five interscreening periods of 2 years each a total of 158 so-called interval cancers were diagnosed. Careful revision of all screening and diagnostic mammograms was executed. Of all interval cancers 26% were 'missed' at the previous screening examination (due to technical or observer error), 16% were radiographically occult at the time of diagnosis and 58% were 'true' interval cancers. Interval cancers were regarded as 'true' when an obvious lesion was observed on the diagnostic mammogram while no suspect signs were seen on the previous screening mammogram. The prevalence of 'missed' cancers did not decline in the course of the screening programme. Radiographically occult tumours were localised, mostly in Wolfe's P2/DY breast parenchyma (83%), 33% were lobular invasive and 25% ductal non-invasive. 'True' interval cancer cases (58%) showed the same overall survival as control breast cancer patients, diagnosed in a non-screening situation. Shortening the screening interval would reduce interval cancer rates and probably further decrease breast cancer mortality in a screened population. However, from the present series of interval cancers 63% would not have been prevented by an annual screening examination. As regards women under age 50 annual screening would still leave 66% of all interval cancers in this age group undetected. Probably more benefit will be gained by searching for new imaging techniques to reduce numbers of 'missed' cancers and to detect lobular invasive and ductal non-invasive cancers in dense breast parenchyma.


*Br. J. Cancer* (1989), **59**, 929–932 © The Macmillan Press Ltd., 1989

### The occurrence of interval cancers in the Nijmegen screening programme

P.H.M. Peeters[1], A.L.M. Verbeek[1], J.H.C.L. Hendriks[2], R. Holland[3], M. Mravunac[4] & G.P. Vooijs[3]

*Departments of [1]Social Medicine, Epidemiology Unit, [2]Radiology, and [3]Pathology, Nijmegen University, Verlengde Groenestraat 75, 6525 EJ Nijmegen and [4]Department of Pathology, Canisius Wilhelmina Hospital, Nijmegen, The Netherlands.*

**Summary** Since January 1975 a population-based screening programme for the early detection of breast cancer has been carried out in the city of Nijmegen. During five interscreening periods of 2 years each a total of 158 so-called interval cancers were diagnosed. Careful revision of all screening and diagnostic mammograms was executed. Of all interval cancers 26% were 'missed' at the previous screening examination (due to technical or observer error), 16% were radiographically occult at the time of diagnosis and 58% were 'true' interval cancers. Interval cancers were regarded as 'true' when an obvious lesion was observed on the diagnostic mammogram while no suspect signs were seen on the previous screening mammogram. The prevalence of 'missed' cancers did not decline in the course of the screening programme. Radiographically occult tumours were localised, mostly in Wolfe's P2/DY breast parenchyma (83%), 33% were lobular invasive and 25% ductal non-invasive. 'True' interval cancer cases (58%) showed the same overall survival as control breast cancer patients, diagnosed in a non-screening situation. Shortening the screening interval would reduce interval cancer rates and probably further decrease breast cancer mortality in a screened population. However, from the present series of interval cancers 63% would not have been prevented by an annual screening examination. As regards women under age 50 annual screening would still leave 66% of all interval cancers in this age group undetected. Probably more benefit will be gained by searching for new imaging techniques to reduce numbers of 'missed' cancers and to detect lobular invasive and ductal non-invasive cancers in dense breast parenchyma.

Within any screening programme for the early detection of breast cancer, women are diagnosed as having so-called interval cancer. These cancers surface among negative screenees before the next scheduled examination would have taken place. Of all breast cancers in a screened population about 20–35% are diagnosed within 2 years after the last screening examination (Verbeek, 1985; Tabár *et al.*, 1987; Moskowitz, 1986; de Waard *et al.*, 1984; Lundgren, 1979). The survival of patients with interval cancers turned out to be just as bad as the survival of patients diagnosed outside screening programmes (Holmberg *et al.*, 1986; Shapiro *et al.*, 1982). This finding would seem to suggest that shortening the screening interval to, say, one year might further decrease breast cancer mortality in a population offered a screening programme. The aim of the present study was to search for more evidence validating such recommendations. The issue of screening frequency is especially relevant for women under age 50, since on the one hand no clear-cut evidence of breast cancer mortality reduction has been demonstrated so far in this age group, and on the other hand relatively high interval cancer rates have been observed (Tabár *et al.*, 1987; Moskowitz, 1987).

### Subjects and methods

All data came from the Nijmegen (150,000 inhabitants) screening programme. This population-based project started in January 1975. Single-view mammography was carried out as the only screening examination every two years. In the first screening round (1975/6) women born in the period 1910–39 ($n=23,000$) were invited. In the subsequent screening rounds women born before 1910 ($n=7,700$) were invited too; in the fifth screening round the cohort of women born in the period 1940–44 ($n=3,900$) was invited. The attendance rate was highest for women under 50 in the first screening round (87%) and lowest for women aged 65 or over in the sixth screening round (31%). Up to December 1986, six screening rounds were performed. In the five interscreening periods 158 breast cancer cases were diagnosed. Two synchronic interval cancers were found. Because some women did not return for screening two years later (at the scheduled two-year interval), some breast cancers were diagnosed in these non-attenders at intervals greater than two years after the negative screening examination. These so-called 'pseudo-interval' cancers were not included in this analysis.

All screening and diagnostic mammograms of the interval cancer cases were carefully reviewed by the radiologist and classified into one of the following three groups. (In five cases the screening or diagnostic mammogram was not available. They were all diagnosed in the early years of the screening project.)

*'Missed' cancers* Forty out of all interval cancers were classified as missed cancers, as due to either technical or observer error.

*Radiographically occult cancers* Twenty-four of all interval cancers were radiologically occult at the time of diagnosis.

*'True' interval cancers* Eighty-nine cancers showed a clear lesion on the diagnostic mammogram and no suspect signs at the preceding screening examination.

It was evaluated whether the number of 'missed' cancers decreased during the 12-year observation period. Interval cancers were compared with breast cancers, detected at one of the five screening rounds, in terms of such radiological and prognostic aspects as Wolfe classification, mammographical tumour size, age at the previous examination, quetelet index, oestrogen receptor positivity and axillary lymph node involvement. To evaluate the prognosis of the 'true' interval cancers the overall survival of these 89 patients was compared with control breast cancer patients in the non-screened population. These control patients were recruited among women who were diagnosed for breast cancer before they received an invitation for a first screening examination.

Distributions of variables in two groups were compared with standard $\chi^2$ tests. Survival curves were computed with

Correspondence: P.H.M. Peeters.
Received 10 July 1988; and in revised form, 24 October 1988.

the actuarial or lifetable method, and the standard errors of these curves computed according to the Greenwood formula. To test equality of survival curves for two groups, the log rank test was used (Mantel, 1966). Adjustment for age on survival was studied with the proportional hazards model (Cox, 1972).

### Results

### *Interval cancers*

Interval cancers occurred more frequently in younger women. In addition to 54 cancers detected by screening in women younger than 50 years, 51 were diagnosed between two consecutive screening examinations. For women aged 50 years or older the figures were 251 and 107 respectively (Tables I and II).

Crude interval cancer rates remained relatively stable over the 12-year period, ranging from 1.57 interval cancers per 1,000 screened women in 2 years' follow-up after the first screening round to 1.61 per 1,000 after the fifth screening round. Even when only regular participants (women attending a screening examination every two years) were included interval cancer rates remained rather stable: 1.63 per 1,000 screenees after the first examination, 1.80 per 1,000 after second, 1.97 per 1,000 after third, 2.21 per 1,000 after fourth and 1.27 per 1,000 after the fifth examination.

The 7-year overall survival for the total group of interval cancers was 74.6% (s.e. ±4.3%).

### *'Missed' cancers*

Of all 153 interval cancers 40 (26%) were classified as 'missed' cancers. There were various reasons why these cancers were not diagnosed at screening examination.

In 14 cases the site of the tumour was outside the image field (technical error) (Table III): twice because of incorrect positioning and 12 times because of an exceptional location of the tumour (high on the chest wall or in the tail of the breast). In 26 cases at revision suspect lesions could be identified in the previous screening mammogram (observer error): in 10 of these those changes were not sufficiently characteristic to warrant a diagnosis of malignancy.

Of all interval cancers diagnosed in the first interval, i.e. between first and second screening round, 16.1% were classified as 'missed' (5/31). For the second, third, fourth and fifth interscreening period these figures were 21.9% (7/32), 44.8% (13/29), 20% (7/35) and 30.8% (8/26), respectively. Although variation is great because of small numbers, these percentages demonstrate no decline in the number of 'missed' cancers since the start of the screening programme.

Patients with 'missed' interval cancers did not differ from screen-detected cases in terms of age at the previous screening examination, Quetelet index, oestrogen receptor positivity or histological tumour type.

They did differ in type of breast parenchyma, although differences in percentages were not statistically significant. (50% P2/DY *vs.* 38%, $P=0.15$.) Also lymph node involvement was more frequently (32% *vs.* 22%, $P=0.20$) and tumour size was significantly larger (95% > 10 mm *vs.* 73%, $P=0.0007$). This may be due to the fact that diagnosis was made on average 11.3 months later. Twenty-three of the 40 'missed' interval cancers were diagnosed within one year.

### *Radiographically occult cancers*

Of the total group of interval cancers 24 (15.7%) were classified as radiologically occult. In 16 of these no signs at all were found on the diagnostic mammogram, while in the other eight mammograms, on careful inspection, very subtle signs, such as a vague density or a slight disturbance of the breast architectural pattern, could be identified. Diagnosis was made on average 13.3 months after the preceding screening examination. Ten of the 24 occult interval cancers occurred within one year after the previous screening examination.

Because radiographically occult breast cancers mark the border of the sensitivity of the mammographical test they were compared with screen-detected cancers and 'true' interval cancers for specific characteristics (Table IV). Women with an occult interval cancer proved to be younger when compared with patients with screen-detected cancers (50% < 50 years *vs.* 18%, $P=0.0001$) and with patients with 'true' interval cancers (50% *vs.* 30%, $P=0.07$). They also had a lower Quetelet index (63% < 25 *vs.* 37% among screen-detected patients, $P=0.02$; *vs.* 33% among patients with 'true' interval cancer, $P=0.008$) which is probably associated with the younger age. There was a striking difference in Wolfe classification and histological type. Of occult tumours 83% were localised in P2/DY breast parenchyma, compared to 38% ($P=0.00001$) among screen-detected and 47% ($P=0.002$) among 'true' interval cancers. Of occult cancers 25% were ductal non-invasive and 33% were invasive lobular; both these rates were higher than those of screen-detected and 'true' interval cancers (10% and 7% among screen-detected, and 6% and 17% among 'true' interval cancers respectively).

### *'True' interval cancers*

On revision of all interval cancers, 89 (58.2%) were not detectable on the screening mammogram. In these patients 7-

**Table I** Number of screened women, number of screen-detected and interval cancer cases (all screenees)

| *Age at screening (years)* | *Round 1 (75/6)* | | *Round 2 (77/8)* | | *Round 3 (79/80)* | | *Round 4 (81/2)* | | *Round 5 (83/4)* | | *Total* | |
|---|---|---|---|---|---|---|---|---|---|---|---|---|
| | *Scr* | *Scr:Int* | *Scr* | *Scr:Int* | *Scr* | *Scr:Int* | *Scr* | *Scr:Int* | *Scr* | *Scr:Int* | *Scr:Int* | *%Int* |
| <50 | 9,681 | 20:15 | 7,165 | 12:9 | 5,508 | 8:10 | 4,276 | 6:12 | 5,911 | 8:5 | 54:51 | 48.6% |
| 50–64 | 9,578 | 53:15 | 8,301 | 32:18 | 7,459 | 21:17 | 7,275 | 16:17 | 7,032 | 26:11 | 148:78 | 34.5% |
| ⩾65 | 443 | 2:1 | 4,321 | 35:9 | 3,662 | 20:3 | 3,540 | 23:6 | 3,227 | 23:10 | 103:29 | 22.0% |
| Total | | | | | | | | | | | 305:158 | 34.1% |

Round number does not necessarily correspond to 'number of examinations', e.g. a woman may have had her first examination in 1977/8, round 2.

**Table II** Distribution of interval cancers according to age, screening round and time interval after the negative screen

| *Age at screening (years)* | *Round 1 (75/6)* | | *Round 2 (77/8)* | | *Round 3 (79/80)* | | *Round 4 (81/2)* | | *Round 5 (83/4)* | |
|---|---|---|---|---|---|---|---|---|---|---|
| | *Int*[1] | *Int*[2] | *Int*[1] | *Int*[2] | *Int*[1] | *Int*[2] | *Int*[1] | *Int*[2] | *Int*[1] | *Int*[2] |
| <50 | 4 | 11 | 4 | 5 | 6 | 4 | 5 | 7 | 4 | 1 |
| 50–60 | 4 | 11 | 8 | 10 | 9 | 8 | 3 | 14 | 5 | 6 |
| 65+ | 1 | 0 | 4 | 5 | 2 | 1 | 1 | 5 | 7 | 3 |

Int[1] = 0–12 months after a negative screen; Int[2] = 13–24 months after a negative screen.

**Table III** Reasons for missed detection on screening mammogram

| | | | |
|---|---|---|---|
| Screening error | Technical error | Incorrect positioning | 2 |
| | | Strange location tumour | 12 |
| | Observer error | Direct signs | 16 |
| | | Less specific signs | 10 |

Direct signs: presence of a mass, malign microcalcifications, nipple retraction, diffuse lymphoedema, skin thickening or spiculation.

Less specific signs: a vague progressive density in a specific area or slight disturbance of the architectural pattern, or slight asymmetry of breast tissue.

year overall actuarial survival was 72.0% (s.e. ±6.1%). In control patients, diagnosed for breast cancer before being eligible for a screening invitation, overall survival was 60.2% (s.e. ±5.2%). Differences in survival curves approached statistical significance (log rank $\chi^2$ 3.2, $P=0.07$). Control patients were older when compared with 'true' interval cancer patients (47% aged 65 years or older *vs.* 21%). Age (continuous) included as an explanatory variable in a proportional hazards model showed a hazard of 0.97 ($P=0.91$) for interval cancer patients compared with control patients. This indicated the hazard to be the same for individuals with 'true' interval cancers as for control patients. The same results were found in proportional hazards models for women below age 50, and for women aged 50 or older. Epidemiological, histological and radiographical features are displayed in Table IV. 'True' interval cancers did not differ from breast cancers in the control group, they only showed less axillary involvement (35.5% *vs.* 58.0%, $P=0.005$). Compared with screen-detected cancers 'true' interval cancers were larger (94% >10 mm *vs.* 73%, $P=0.02$). Diagnosis was made on average 15.2 months after the previous screening examination. Thirty-six per cent of 'true' interval cancers were diagnosed within one year after the screening examination.

### Discussion

The results of breast cancer screening projects such as the HIP-trial in the United States (Shapiro *et al.*, 1982) the DOM-project in Utrecht (Collette *et al.*, 1984), the Nijmegen screening project (Verbeek *et al.*, 1984), the Italian project (Palli *et al.*, 1986) and the Swedish trial (Tabár *et al.*, 1985*a*) show a considerable reduction of breast cancer mortality. But even though early detection and early treatment are no longer disputed as being beneficial, some unsolved problems remain. One of the major problems one faces in a breast cancer screening project is the number of interval cancers. In the Nijmegen programme, where a screening examination was performed every 2 years, crude interval cancer rates remained relatively stable over the 12-year period. Other studies showed similar interval cancer rates. (Baker, 1982; Frisell *et al.*, 1987; Tábar *et al.*, 1985*b*). Interval cancers occurred more frequently among women under 50 years of age, when compared to women aged 50 or older. For younger women the ratio between interval cancers and screen-detected cancers was about 1:1 while this was about 1:2.5 for women aged 50 or older.

Previous studies (Holland *et al.*, 1982; Newsome & McLelland, 1986; Martin *et al.*, 1979; van Rosen *et al.*, 1985) showed one third of all interval cancers to be missed at a preceding screening examination due to either technical or observer error. In the present study 26% of interval cancers were missed. This percentage did not decline in the course of the programme. To some extent this may have been caused by the entry of new cohorts of women into the screening project. In the Nijmegen programme women born in the period 1940–44 were not invited for a screening examination until 1983/4. Reading mammograms of these young women is difficult due to the high density of the breast parenchyma. Some of the missed cancers, however, were due to non-specific changes, such as a vague progressive density or a slightly disturbed architectural pattern. Referral of such lesions for further clinical evaluation probably would have resulted in a large number of false-positive screening results. From a total of 153 interval cancers, 16% were radiographically occult at the time of diagnosis. Occult cancers are clinically detectable before they show mammographically suspect signs. During growth they tend to remain obscured by dense P2/DY breast parenchyma. Occult cancers often were of lobular invasive or ductal non-invasive histological type. In other studies about 5–7% of breast cancer patients were reported to have negative mammograms. Patients were younger compared with all other breast cancer patients (Burns *et al.*, 1979; Cahill *et al.*, 1981). Here the limits of modern mammography have been reached since in dense breast parenchyma these types of breast cancers probably cannot be visualised (Holland *et al.*, 1983).

Of all interval cancers 58% showed no suspect lesions on a previous screening mammogram, while they were visible at the time of diagnosis. They were either masked in some way at the previous examination or were newly grown, which implies a high growth rate. A similar percentage of 'true' interval cancers (52%) was found in the Stockholm screening programme (Frisell *et al.*, 1987). Although in patients with 'true' interval cancers axillary lymph node involvement was statistically significantly less frequent (36% *vs.* 58%) when compared with control breast cancer patients, 7-year overall survival was equal. These results are identical to those

**Table IV** Percentages of epidemiological, histological and radiological aspects of interval cancers (i.e. 'missed' cancers, radiographically occult cancers and 'true' interval cancers), screen-detected cancers and cancers of control patients (i.e. with a diagnosis of breast cancer before any screening invitation)

| Factor | *'Missed' cancers (n=40)* | *Radiographically occult cancers (n=24)* | *'True' interval (n=89)* | *Screen-detected cancers (n=305)* | *Control patients (n=127)* |
|---|---|---|---|---|---|
| Age <50 years[a] | 27.5 | 50.0 | 30.3 | 17.7 | 38.6[h] |
| Quetelet index <25[b] | 37.5 | 62.5 | 32.6 | 37.4 | –[i] |
| Oestrogen receptor pos.[c] | 75.7 | 64.3 | 65.8 | 80.0 | 62.5 |
| Wolfe P2/DY | 50.0 | 83.3 | 47.2 | 38.0 | 50.4 |
| Axillary node involvement[d] | 32.4 | 28.6 | 35.5 | 22.4 | 58.0 |
| Tumour size <10 mm[e] | 5.0 | – | 5.6 | 27.2 | 10.4 |
| Histology: DCIS[f] | – | 25.0 | 5.6 | 9.9 | 3.2 |
| Duct invasive | 74.4 | 41.7 | 66.3 | 75.0 | 81.1 |
| Lobular invasive | 12.8 | 33.3 | 16.9 | 6.9 | 9.5 |
| Other[g] | 12.8 | – | 11.2 | 8.2 | 6.3 |

[a]Age at screening examination; [b]Quetelet index = $kg\,m^{-2}$; [c]Oestrogen receptor positive $\geqslant 10\,fmol\,mg^{-1}$; [d]In the early years of the screening programme it was neither a national nor a local practice to remove the axillary lymph nodes for histologic examination; [e]Size of the tumour on the mammogram at diagnosis; [f]DCIS = duct carcinoma *in situ*; [g]Other = tubular carcinoma, medullary carcinoma, papillary carcinoma; [h]Age = age at diagnosis; [i]Not known for control patients.

reported by others (Holmberg *et al.*, 1986; Shapiro *et al.*, 1982).

It is often argued that shortening the screening interval would reduce the number of interval cancers (Tabár *et al.*, 1987; Moskowitz & Gartside, 1982). Especially the high proportion of interval cancers occurring in women under age 50 is reported to cause the absence of a clear reduction in mortality in women from this age-group, after participating in a screening programme. More frequent screening will probably not affect the number of 'missed' cancers, since the same error rates of about 30% are found in studies with different screening intervals (Baker, 1982; Holland *et al.*, 1982; Newsome & McLelland, 1986; Martin *et al.*, 1979). In the Nijmegen project the prevalence of 'missed' interval cancers did not decrease during the progress of the programme. More frequent screening will certainly not improve the detection of radiographically occult breast cancers, it can only influence the 'true' interval cancer group of 58%. However, since 32 of these 89 interval cancers occurred within one year after the previous screening examination an annual screening examination would still leave 40 ('missed' cancers) plus 24 (radiographically occult cancers) plus 32 ('true' interval cancers occurring within one year) undetected. So from the present series of 153 interval cancers, 96 (63%) would not have been prevented by more frequent screening. As regards women under age 50, from a total of 51 interval cancers (50, one missing) 11 were 'missed' at the previous screening examination, 12 were radiographically occult and 10 of the 'true' interval cancers occurred within one year after the preceding screening examination. For this age-group shortening the screening interval from 2 years to 1 year would prevent 34% of interval cancers. This age-group would probably benefit more from the development of new imaging techniques to detect specific lobular invasive and ductal non-invasive cancers in dense breast tissue.

We thank Ms H. Rijken (Department of Radiology), Ms I. Sybenga-van der Steen, Ms L. Traa and E. Brummelkamp (Department of Social Medicine) for data analysis, processing and gathering, and F. de Groot for his comments. This study was partly supported by the Praeventiefonds.

### References

BAKER, L.H. (1982). Breast Cancer Detection Demonstration Project: Five-year summary report. *Cancer*, **32**, 194.

BURNS, P.E., GRACE, M.G.A., LEES, A.W. & MAY, C. (1979). False negative mammograms causing delay in breast cancer diagnosis. *J. Can. Assoc. Radiol.*, **30**, 74.

CAHILL, C.J., BOULLEZ, P.S., GIBBS, N.M. & PRICE, J.H. (1981). Features of mammographically negative breast tumours. *Br. J. Surg.*, **68**, 882.

COLLETTE, H.J.A., DAY, N.E., ROMBACH, J.J. & DE WAARD, F. (1984). Evaluation of screening for breast cancer in a non-randomised study (the DOM project) by means of a case-control study. *Lancet*, **i**, 1224.

COX, D.R. (1972). Regression models and life tables (with discussion). *J. Stat. Soc.*, **34**, 187.

FRISELL, J., EKLUND, G., HELLSTRÖM, L. & SOMELL, A. (1987). Analysis of interval breast carcinomas in a randomized screening trial in Stockholm. *Breast Cancer Res. Treat.*, **9**, 219.

HOLLAND, R., MRAVUNAC, M., HENDRIKS, J.H.C.L. & BEKKER, B.V. (1982). So-called interval cancer of the breast. Pathologic and radiologic analysis of sixty-four cases. *Cancer*, **49**, 2527.

HOLLAND, R., HENDRIKS, J.H.C.L. & MRAVUNAC, M. (1983). Mammographically occult breast cancer. A pathologic and radiologic study. *Cancer*, **52**, 1810.

HOLMBERG, L.H., ADAMI, H.O., TABÁR, L. & BERGSTROM, R. (1986). Survival in breast cancer diagnosed between mammographic screening examinations. *Lancet*, **ii**, 27.

LUNDGREN, B. (1979). Efficiency of single-view mammography: Rate of interval cancer cases. *J. Natl Cancer Inst.*, **62**, 799.

MANTEL, N. (1966). Evaluation of survival data and two new rank order statistics arising in its consideration. *Cancer Chemother. Rep.*, **50**, 163.

MARTIN, J.E., MOSKOWITZ, M. & MILBRATH, J.R. (1979). Breast cancer missed by mammography. *A.J.R.*, **132**, 737.

MOSKOWITZ, M. & GARTSIDE, P.S. (1982). Evidence of breast cancer mortality reduction: Aggressive screening in women under age 50. *A.J.R.*, **138**, 911.

MOSKOWITZ, M. (1986). Breast cancer: Age-specific growth rates and screening strategies. *Radiology*, **161**, 37.

MOSKOWITZ, M. (1987). Cost-benefit determinations in screening mammography. *Cancer*, suppl. **60**, 1680.

NEWSOME, J.F. & MCLELLAND, R. (1986). A word of caution concerning mammography. *J.A.M.A.*, **255**, 528.

PALLI, D., ROSSELLI, DEL TURCO, M., BUIATTI, E. & 4 others (1986). A case-control study of the efficacy of a non-randomized breast cancer screening program in Florence (Italy). *Int. J. Cancer*, **38**, 501.

ROSEN VON, A., ERHARDT, K., HELLSTRÖM, L., SOMELL, A. & AUER, G. (1985). Assessment of malignancy potential in so-called interval mammary carcinomas. *Breast Cancer Res. Treat.*, **6**, 221.

SHAPIRO, S., VENET, W., STRAX, PH., VENET, L. & ROESER, R. (1982). Ten-to-fourteen year effect of screening on breast cancer mortality. *J. Natl Cancer Inst.*, **69**, 349.

TABÁR, L., FAGERBERG, G., DAY, N.E. & HOLMBERG, L. (1987). What is the optimum interval between mammographic screening examinations? An analysis based on the latest results of the Swedish two-county breast cancer screening trial. *Br. J. Cancer*, **55**, 547.

TABÁR, L., FAGERBERG, C.J.G., GAD, A. & 9 others (1985*a*). Reduction in mortality from breast cancer after mass screening with mammography. *Lancet*, **i**, 829.

TABÁR, L., GAD, A., HOLMBERG, L. & LJUNGQUIST, U. (1985*b*). Significant reduction in advanced breast cancer. Results of the first seven years of mammography screening in Kopparberg, Sweden. *Diagn. Imag. Clin. Med.*, **54**, 158.

VERBEEK, A.L.M., HENDRIKS, J.H.C.L., HOLLAND, R., MRAVUNAC, M., STURMANS, F. & DAY, N.E. (1984). Reduction of breast cancer mortality through mass screening with modern mammography. First results of the Nijmegen project, 1975–1981. *Lancet*, **i**, 1222.

VERBEEK, A.L.M. (1985). Population screening for breast cancer in Nijmegen. An evaluation of the period 1975–1982. Thesis, Nijmegen University.

DE WAARD, F., COLLETTE, H.J.A., ROMBACH, J.J., BAANDERS, VAN HALEWIJN, E.A. & HONING, C. (1984). The DOM project for the early detection of breast cancer, Utrecht, The Netherlands. *J. Chronic Dis.*, **37**, 1.